# Protective efficacy of statins in patients with *Klebsiella pneumoniae* bloodstream infection

**DOI:** 10.3389/fcimb.2022.1087701

**Published:** 2023-01-05

**Authors:** Qian Xu, Beiwen Zheng, Ping Shen, Yonghong Xiao

**Affiliations:** ^1^ Laboratory Medicine Center, Department of Transfusion Medicine, Zhejiang Provincial People's Hospital, Affiliated People's Hospital, Hangzhou Medical College, Hangzhou, Zhejiang Province, China; ^2^ State Key Laboratory for Diagnosis and Treatment of Infectious Diseases, National Clinical Research Center for Infectious Diseases, Collaborative Innovation Center for Diagnosis and Treatment of Infectious Diseases, The First Affiliated Hospital, Zhejiang University, School of Medicine, Hangzhou, Zhejiang Province, China; ^3^ Jinan Microecological Biomedicine Shandong Laboratory, Jinan, Shandong Province, China

**Keywords:** *Klebsiella pneumoniae*, bloodstream infection, 28-day mortality, septic shock, statins

## Abstract

**Background:**

Patients with bloodstream infection of *Klebsiella pneumoniae* (BSI-KP) have a high risk of death and septic shock. This study aims to identify the risk factors for mortality and severity in patients of BSI-KP.

**Methods:**

Data of BSI-KP patients were extracted from the MIMIC IV (Medical Information Mart for Intensive Care IV) database, and patients infected with only *K. pneumoniae* in blood were included in this study. The risk factors of 28-day mortality and septic shock in BSI-KP patients were analyzed, respectively.

**Results:**

A total of 279 patients enrolled and the all-cause 28-day mortality rate was 11.8%. The use of statins (OR 0.220, 95% CI 0.060-0.801, p = 0.022) and quinolones (OR 0.356, 95% CI 0.143-0.887, p = 0.027) were both independent protective factors for death within 28 days, while the use of vasoactive drugs (OR 7.377, 95% CI 1.775-30.651, p = 0.006) was a risk factor. Besides, pulmonary disease (OR 2.348, 95% CI 1.126-4.897, p = 0.023), bleeding and coagulation disorders (OR 3.626, 95% CI 1.783-7.372, p < 0.001), respiratory failure (OR 2.823, 95% CI 0.178-6.767, p = 0.020) and kidney dysfunction (OR 2.450, 95% CI 1.189-5.047, p = 0.015) were independent risk factors for patients suffered from septic shock while hypertension was a protective one. The receiver operating characteristic (ROC) curves could well predict the risk of death within 28-day (area under ROC = 0.855, 95% CI = 0.796–0.914, p < 0.001) and septic shock (AUROC = 0.815, 95% CI = 0.755–0.874, p < 0.001) in patients with BSI-KP.

**Conclusion:**

The use of statins could decrease the risk of 28-day mortality in patients of BSI-KP. The risk factor-based prediction model provided evidence for drug treatment in BSI-KP patients. Paying more attention to the strategy of drug treatment will be an optimal way to improve patient’s outcome in clinical practice.

## Introduction

*Klebsiella pneumoniae* is a Gram-negative, encapsulated, facultatively anaerobic bacteria which is one of the most important opportunistic pathogens in hospitals all over the world (Wyres et al., 2020; Chang et al., 2021). With the widespread around the world, the infection of *K. pneumoniae* has caused huge health and economic burden to mankind in the past decade. In 2017, the World Health Organization included it in the list of dominant pathogens, emphasizing its extremely important position in public health issues (World Health Organization, 2017).

In humans, *K. pneumoniae* often colonizes the nasal and digestive tract without causing any symptomatic disease. However, the colonization can turn into a bloodstream infection of *K. pneumoniae* (BSI-KP) when the host immunity fails to control the pathogen growth. As a large-scale multicenter epidemiological study and in-depth genomic analysis in China demonstrated, BSI-KP has a considerable prevalence and high mortality worldwide (Jia et al., 2021). It is reported that the case fatality rate of BSI is between 21% and 69% (Zhang et al., 2020). Clinicians give great attention to BSI-KP because it can easily cause sepsis/septic shock in patients that often lead to multiple organ dysfunction syndromes (MODS) and death, especially encountered with hyper-virulent strains. Furthermore, BSI-KP can affect the patient's prognosis by increasing the length of hospital stay, treatment cost, and complications after discharge. Therefore, it is an urgent and valuable task for us to explore the risk predictors in patients with BSI-KP.

Statin is a kind of commonly used drug in clinical practice and have both lipid-lowering and anti-inflammatory properties. Nearly twenty years ago, Almog et al. (Almog, 2003) hypothesized that statins have a strong protective effect against sepsis by virtue of diverse anti-inflammatory effects that are independent of their lipid-lowering ability. Five years ago, Lee et al. (Lee et al., 2018) found that statins potentially decreased the 30-day mortality in sepsis patients from a large population.Recently, it was found that prior statin treatment take the positive effect in patients with COVID-19 and type 2 diabetes infection (Cheng et al., 2019; Davoudi et al., 2021). During the past two decades, a few researches was focused on the evidence of the statins use improving the outcome of patients with different infections, but are still need to be strengthened.

Patients with BSI-KP have a relatively high risk of poor outcomes, and the situation of CRKP infection often emerged after treating with antibiotics (Fair and Tor, 2014). There were a series of studies on the risk factors of mortality in patients infected with carbapenem-resistant *K. pneumoniae* (CRKP) but few was focused on the susceptible strains which accounted for majority episodes in clinical settings (Jia et al., 2021). The database of MIMIC IV (Medical Information Mart for Intensive Care IV) includes clinical data of patients with BSI-KP during an eleven-year period, and most of the *K. pneumoniae* strains were susceptible to carbapenems. In this study, based on the MIMIC IV database, we aimed to find effective factors especially the medication factors such as antibiotics and statins, to predict the risk of death and severity, which would provide the evidence for clinical practice in reducing the mortality and severity in BSI-KP patients.

## Methods

### Data source

The data in this retrospective study were extracted from the MIMIC IV (Johnson et al., 2021), which is an extensive, freely-available database comprising de-identified health-related data from patients who were admitted to a tertiary academic medical center in Boston, MA, USA. It contains information between 2008-2019 for each patient while they were in the hospital: laboratory measurements, medications administered, vital signs documented, and so on. The patients’ information was anonymized, and thus the need for patients’ informed consent was waived for this study. All data in this study were extracted by the first author (certification number: 37986401), who passed the Collaborative Institutional Training Initiative examination and achieved access to the database for data extraction. The use of the database was approved by the institutional review boards of the Massachusetts Institute of Technology. All data analysis and reporting has been performed in accordance with institutional guidelines and regulations.

### Study population

A total of 6,189 records of positive blood culture with *K. pneumoniae* in the MIMIC IV database from 2008 to 2019 were included in this work. For a collection of patients infected, the duplicated hospitalization and culture record of each patient and culture time three days before admission time or after discharge time were both excluded. For patients who had admission more than once, only data of the first admission was included. For patients who had positive culture results more than once, only data of the first culture was included. Then patients infected with only *K. pneumoniae* in blood were screened out by exclusion criteria: (1). patients with missing key data; (2). non-adult patients (Age<18); (3). patients with other positive pathogens in blood.

### Variable extraction

Data from the MIMIC-IV database that were considered baseline characteristics during the hospitalization listed as follows: age, gender, comorbidities (cardiovascular disease, pulmonary disease, cerebrovascular disease, encephalopathy, hypertension, diabetes mellitus, solid tumor, hematological malignancy, venous thromboembolism, bleeding, and coagulation disorders, hyperlipidemia, etc.), personal history (drinking, smoking, etc.), Charlson comorbidity index (CCI) and days of hospital stay. Besides, the occurrence of invasive operation, vasoactive agent use, and information of antimicrobial strategy were also explored. Data extraction was accomplished using BigQuery, an online database management system that associated with the MIMIC IV data (http://console.cloud.google.com/bigquery).

### Definitions of variables

In an appropriate antimicrobial strategy, empirical therapy was defined as the antimicrobials administered before a susceptibility report was available, and combination therapy was defined as the administration of more than one antibiotic. Community-acquired here meant the source of the bacteria and was defined as the culture time fallen between 3 days before admission and two days after admission. Among all patients, the three most common and significant categories in antibiotics varied from β-lactamase/β-lactamase inhibitors, carbapenems, and quinolones. Emergency admission represented the patients admitted from the emergency department. The invasive line included all blood-related pipelines and ventilation comprised of invasive and non-invasive mechanical types. The outcome was determined as all-cause death at 28 days after admission.

### Statistical analysis

Continuous variables were expressed as mean ± standard deviation (SD) or median [interquartile range (IQR)] as appropriate. Categorical variables were expressed as percentages (%). The Chi-square test or Fisher's exact test was used to test comparisons between groups for categorical variables. In contrast, the t-test or Mann–Whitney U test was used to compare continuous variables, as appropriate. The risk factors were analyzed using binary logistic regression, and age, sex, and the univariable with p < 0.05 were included in the multivariate logistic regression models. Odds ratios (ORs) and 95% confidential intervals (CIs) were calculated. All p values were two-tailed, and p < 0.05 was considered statistically significant. The linear relationship between the logit conversion values of continuous independent variables and dependent variables was confirmed, and no multicollinearity was found between independent variables before the analysis of logistic regression. A risk prediction model for mortality was established based on the binary logistic regression equation. The model's prediction performance and fitting degree were evaluated by the receiver operating characteristic (ROC) curve and Hosmer-Lemeshow test, respectively. All statistical analysis was performed using the SPSS version 24.0, and *p*<0.05 was considered statistically significant. Graphs in this study were created by GraphPad Prism version 7.04.

## Results

### Patients’ characteristics

**Figure 1:**
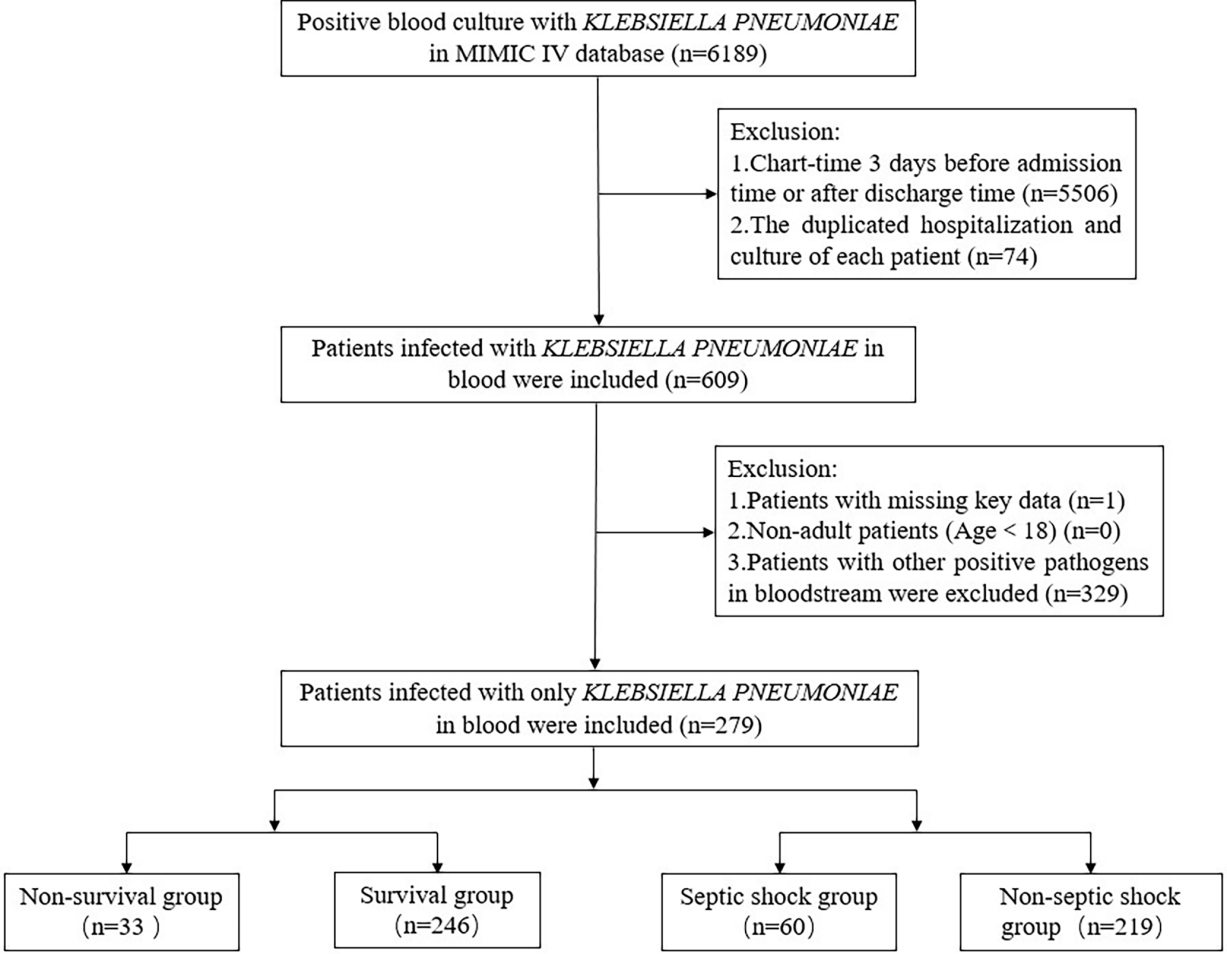
Flowchart of patients’ selection.

There were 279 patients with BSIs caused by only *K. pneumoniae* in the MIMIC IV database (**Figure 1**). The demographic and clinical characteristics of all patients are listed in **Table 1**. A total of 156 (55.9%) patients are males, and the median age was 67 (IQR: 56–77) years. Hypertension (57.0%) was the most prevalent comorbidity, followed by kidney dysfunction (51.3%), cardiovascular disease (45.5%), solid tumor (45.2%) and pulmonary disease (43.7%). The median CCI was 6 (IQR: 4–8). Besides, the number of community-acquired was 204 (73.1%), and the emergency type of admission was 222 (79.6%). In addition, the history of smoking (32.6%), drinking (7.9%), surgery and trauma (60.6%), and hospitalization (64.2%) of each patient were also summarized. The patients' 28-day all-cause mortality and septic shock incidence were 11.8% and 21.5%, respectively.

**Table 1:** The demographic and clinical characteristics in non-survival and survival groups of 279 patients with BSI-KP.

Variables, n (%)	Total (n=279)	Non-survival group (n=33)	Survival group (n=246)	P-value
Age, years (IQR)	67 (56-77)	69 (55-80)	66 (56-77)	0.429
Male	156 (55.9)	21 (63.6)	135 (54.9)	0.341
Comorbidity				
Cardiovascular disease	127 (45.5)	18 (54.5)	112 (44.3)	0.268
Pulmonary disease	122 (43.7)	22 (66.7)	100 (40.7)	**0.005**
Cerebrovascular disease	37 (13.3)	2 (6.1)	35 (14.2)	0.276
Encephalopathy	70 (25.1)	9 (27.3)	61 (24.8)	0.758
Diabetes mellitus	107 (38.4)	10 (30.3)	97 (39.4)	0.311
Hypertension	159 (57.0)	12 (36.4)	147 (59.8)	**0.011**
Solid tumor	126 (45.2)	11 (33.3)	115 (46.7)	0.146
Hematological malignancy	22 (7.9)	4 (12.1)	18 (7.3)	0.309
VTE	43 (15.4)	4 (12.1)	39 (15.9)	0.577
Bleeding and coagulation disorders	73 (26.2)	16 (48.5)	57 (23.2)	**0.002**
Hyperlipidemia	109 (39.1)	6 (18.2)	103 (41.9)	**0.009**
Heart failure	48 (17.2)	8 (24.2)	40 (16.3)	0.254
Respiratory failure	44 (15.8)	15 (45.5)	29 (11,8)	**<0.001**
Kidney dysfunction	143 (51.3)	22 (66.7)	121 (49.2)	0.059
CCI, median (IQR)	6 (4-8)	6 (5-9)	6 (4-8)	0.195
Past history				
Smoking	91 (32.6)	9 (27.3)	82 (33.3)	0.486
Drinking	22 (7.9)	5 (15.2)	17 (6.9)	0.157
Surgery and trauma	169 (60.6)	17 (51.5)	152 (61.8)	0.257
Prior hospitalization	179 (64.2)	16 (48.5)	163 (66.3)	**0.046**
Epidemiology				
Community-acquired	204 (73.1)	23 (69.7)	181 (73.6)	0.637
Emergency admission	222 (79.6)	28 (84.8)	194 (78.9)	0.423
Invasive operation				
Invasive line	87 (31.2)	25 (75.8)	62 (25.2)	**<0.001**
Ventilation	101 (36.2)	25 (75.8)	76 (30.9)	**<0.001**
Appropriate antimicrobial strategy				
Empirical treatment	45 (16.1)	6 (18.2)	39 (15.9)	0.733
Combination therapy	224 (80.3)	27 (81.8)	197 (80.1)	0.814
Treated with BLBLIs	144 (51.6)	22 (66.7)	122 (49.6)	0.065
Treated with Carbapenems	58 (20.8)	8 (24.2)	50 (20.3)	0.603
Treated with Quinolones	161 (57.7)	13 (39.4)	148 (60.2)	**0.023**
More than two categories of antibiotics	111 (39.8)	14 (42.4)	97 (39.4)	0.741
Related agent use				
Vasoactive agent use	74 (26.5)	25 (75.8)	49 (19.9)	**<0.001**
Statins use	91 (32.6)	5 (15.2)	86 (35.0)	**0.023**
Outcome				
Septic shock	60 (21.5)	18 (54.5)	42 (17.1)	**<0.001**
Days of hospital stay (IQR)	6.3 (4.0-11.3)	6.0 (1.3-12.2)	6.3 (4.2-11.3)	0.059

IQR, interquartile range; CCI, Charlson comorbidity index; VTE, Venous thromboembolism; BLBLI, beta-lactam-beta-lactamase inhibitor; OR, odds ratio; CI, confidence interval. The P-values less than 0.05 are in bold.

### Predictors for 28-day mortality

Of the 279 patients, 33 were not survived (non-survival group) and 246 survived (survival group) on day of 28. The univariate analysis results indicated the variables associated with 28-day mortality as followed (**Table 1**): pulmonary disease (p=0.005), hypertension (p = 0.011), bleeding and coagulation disorders (p =0.002), hyperlipidemia (p = 0.009), respiratory failure (p < 0.001), invasive line (p < 0.001), ventilation (p < 0.001), prior hospitalization (p = 0.046), treated with quinolones (p = 0.023), vasoactive agent use (p < 0.001), statins use (p = 0.023), septic shock (p < 0.001).

Co-operated with age and gender in multivariate analysis (**Table 2**), the independent risk factor for 28-day mortality was vasoactive agent use (OR 7.377, 95% CI 1.775-30.651, p = 0.006). By contrast, age (OR 0.965, 95% CI 0.932-0.999, p =0.041), statins use (OR 0.220, 95% CI 0.060-0.801, p =0.022) and treated with quinolones (OR 0.356, 95% CI 0.143-0.887, p = 0.027) were all protective factors for 28-day mortality in patients of BSI-KP.

**Table 2:** Predictors for 28-day mortality in non-survival and survival group by univariate and multivariate analysis.

Variables	Univariate		Multivariate	
	OR (95%CI)	P-value	OR (95%CI)	P-value
Age, years	–	0.429	0.965 (0.932-0.999)	**0.041**
Male	1.439 (0.678-3.053)	0.341	1.786 (0.700-4.552)	0.225
Pulmonary disease	2.920 (1.356-6.289)	0.005	1.274 (0.463-3.508)	0.639
Hypertension	0.385 (0.181-0.818)	0.011	0.719 (0.272-1.901)	0.506
Bleeding and coagulation disorders	3.121 (1.483-6.568)	0.002	1.272 (0.464-3.489)	0.640
Hyperlipidemia	0.309 (0.123-0.774)	0.009	0.368 (0.112-1.204)	0.098
Respiratory failure	6.236 (2.838-13.701)	<0.001	2.431 (0.725-8.151)	0.150
Invasive line	9.274 (3.978-21.624)	<0.001	1.703 (0.384-7.554)	0.484
Ventilation	6.990 (3.015-16.205)	<0.001	1.347 (0.338-5.367)	0.673
Hospitalization	0.479 (0.230-0.997)	0.046	1.378 (0.511-3.717)	0.527
Treated with Quinolones	0.430 (0.205-0.905)	0.023	0.356 (0.143-0.887)	**0.027**
Vasoactive agent use	12.564 (5.341-29.554)	<0.001	7.377 (1.775-30.651)	**0.006**
Statins use	0.332 (0.124-0.891)	0.023	0.220 (0.060-0.801)	**0.022**
Septic shock	5.829 (2.722-12.481)	<0.001	0.903 (0.285-2.860)	0.863

OR, odds ratio; CI, confidence interval. The P-values less than 0.05 are in bold.

### Predictors of septic shock

Among the study population, there were 60 and 219 patients in septic shock group and non- septic shock group, respectively. As demonstrated in **Table 3**, the univariate analysis results indicated the variables associated with sepsis/septic shock as followed: pulmonary disease (p < 0.001), hypertension (p = 0.016), bleeding and coagulation disorders (p < 0.001), respiratory failure (p < 0.001), kidney dysfunction (p = 0.001), prior hospitalization (p = 0.004).

**Table 3:** The demographic and clinical characteristics in septic shock and non-septic shock of 279 patients with BSI-KP.

Variables, n (%)	Total (n=279)	Septic shock group (n=60)	Non-septic shock group (n=219)	P value
Age, years median (IQR)	67 (56-77)	67 (53-81)	67 (56-78)	0.769
Male	156 (55.9)	28 (46.7)	128 (58.4)	0.103
Comorbidity				
Cardiovascular disease	127 (45.5)	29 (48.3)	98 (44.7)	0.621
Pulmonary disease	122 (43.7)	39 (65.0)	83 (37.9)	**<0.001**
Cerebrovascular disease	37 (13.3)	5 (8.3)	32 (14.6)	0.204
Encephalopathy	70 (25.1)	16 (26.7)	54 (24.7)	0.750
Diabetes mellitus	107 (38.4)	27 (45.0)	80 (36.5)	0.232
Hypertension	159 (57.0)	26 (43.3)	133 (60.7)	**0.016**
Solid tumor	126 (45.2)	23 (38.3)	103 (47.0)	0.230
Hematological malignancy	22 (7.9)	5 (8.3)	17 (7.8)	1.000
VTE	43 (15.4)	7 (11.7)	36 (16.4)	0.364
Bleeding and coagulation disorders	73 (26.2)	31 (51.7)	42 (19.2)	**<0.001**
Hyperlipidemia	109 (39.1)	19 (31.7)	90 (41.1)	0.185
Heart failure	48 (17.2)	12 (20.0)	36 (16.4)	0.517
Respiratory failure	44 (15.8)	23 (38.3)	21 (9.6)	**<0.001**
Kidney dysfunction	143 (51.3)	42 (70.0)	101 (46.1)	**0.001**
CCI, median (IQR)	6 (4-8)	6 (4-8)	6 (4-9)	0.912
Past history				
Smoking	91 (32.6)	15 (25.0)	76 (34.7)	0.155
Drinking	22 (7.9)	5 (8.3)	17 (7.8)	1.000
Surgery and trauma	169 (60.6)	39 (65.0)	130 (59.4)	0.428
Prior hospitalization	179 (64.2)	29 (48.3)	150 (68.5)	**0.004**
Epidemiology				
Community-acquired	204 (73.1)	38 (63.3)	166 (75.8)	0.054
Emergency admission	222 (79.6)	49 (81.7)	173 (79.0)	0.649
**Invasive operation**				
Invasive line	87 (31.2)	50 (83.3)	37 (16.9)	**<0.001**
Ventilation	101 (36.2)	48 (80.0)	53 (24.2)	**<0.001**
Appropriate antimicrobial strategy				
Empirical treatment	45 (16.1)	9 (15.0)	36 (16.4)	0.788
Combination therapy	224 (80.3)	53 (88.3)	171 (78.1)	0.077
Treatment with BLBLIs	144 (51.6)	40 (66.7)	104 (47.5)	**0.008**
Treatment with Carbapenems	58 (20.8)	17 (28.3)	41 (18.7)	0.104
Treatment with Quinolones	161 (57.7)	32 (53.3)	129 (58.9)	0.439
More than two categories of antibiotics	111 (39.8)	14 (42.4)	97 (39.4)	0.741
Related agent use				
Vasoactive agent use	74 (26.5)	49 (81.7)	25 (11.4)	**<0.001**
Statins use	91 (32.6)	17 (28.3)	74 (33.8)	0.424
Outcome				
death within 28 days	33 (11.8)	18 (30.0)	15 (6.8)	**<0.001**
Days of hospital stay (IQR)	6.3 (4.0-11.3)	6.5 (3.9-15.5)	6.2 (4.0-10.8)	0.423

IQR, interquartile range; CCI, Charlson comorbidity index; VTE, Venous thromboembolism; BLBLI, beta-lactam-beta-lactamase inhibitor; OR, odds ratio; CI, confidence interval. The P-values less than 0.05 are in bold.

As the results of multivariate analysis, pulmonary disease (OR 2.348, 95% CI 1.126-4.897, p = 0.023), bleeding and coagulation disorders (OR 3.626, 95% CI 1.783-7.372, p < 0.001), respiratory failure (OR 2.823, 95% CI 0.178-6.767, p = 0.020) and kidney dysfunction (OR 2.450, 95% CI 1.189-5.047, p = 0.015) were independent risk factors while male (OR 0.333, 95% CI 0.161-0.688, p =0.003) and hypertension (OR 0.363, 95% CI 0.180-0.733, p = 0.005) was protective factors for patients suffered from septic shock (**Table 4**).

**Table 4:** Predictors for 28-day mortality in septic shock and non-septic shock group by univariate and multivariate analysis.

Variables, n (%)	Univariate		Multivariate	
	OR (95%CI)	P-value	OR (95%CI)	P-value
Age, years	–	0.769	0.992 (0.968-1.016)	0.497
Male	0.622 (0.350-1.104)	0.103	0.333 (0.161-0.688)	**0.003**
Pulmonary disease	3.043 (1.676-5.526)	<0.001	2.348 (1.126-4.897)	**0.023**
Hypertension	0.494 (0.277-0.882)	0.016	0.363 (0.180-0.733)	**0.005**
Bleeding and coagulation disorders	4.505 (2.453-8.274)	<0.001	3.626 (1.783-7.372)	**<0.001**
Respiratory failure	5.861 (2.946-11.660)	<0.001	2.823 (1.178-6.767)	**0.020**
Kidney dysfunction	2.726 (1.477-5.031)	0.001	2.450 (1.189-5.047)	**0.015**
Prior hospitalization	0.430 (0.241-0.769)	0.004	0.601 (0.300-1.204)	0.151

OR: odds ratio; CI: confidence interval. The P-values less than 0.05 are in bold.

### Comparison of antimicrobial susceptibility

**Figure 2:**
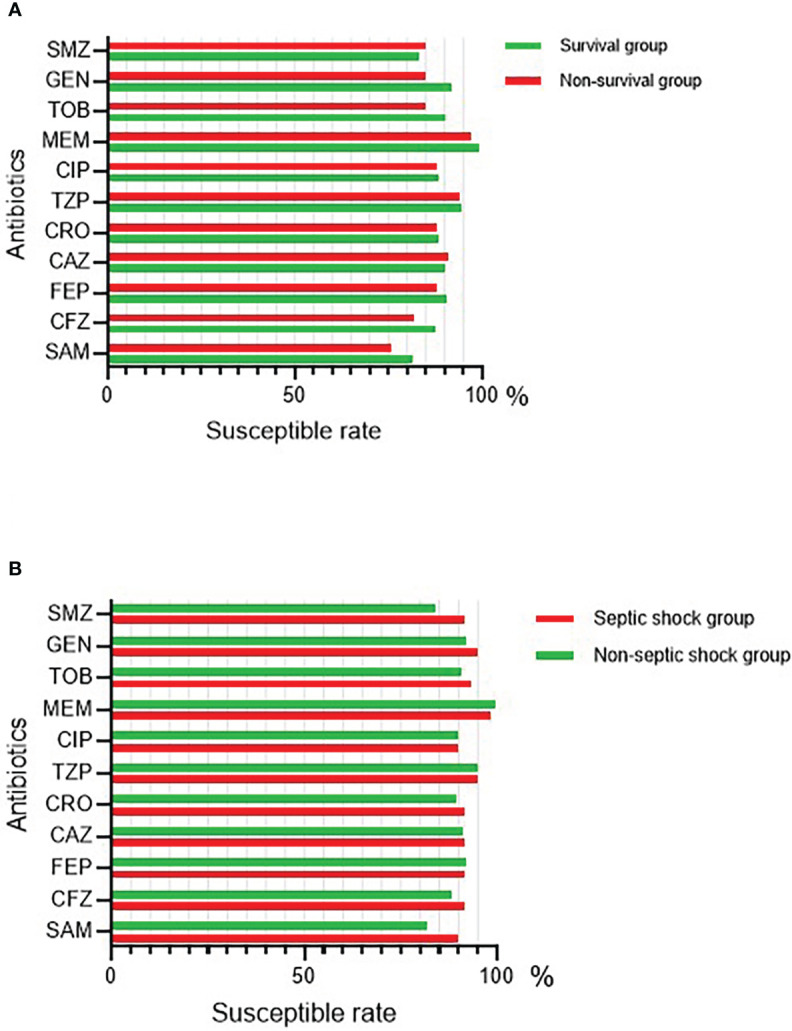
The antimicrobial susceptibility of BSI-KP in 279 patients. **(A)** The antimicrobial susceptibility of BSI-KP in survival and non-survival group. **(B)** The antimicrobial susceptibility of BSI-KP in septic shock and non-septic shock group. SAM, ampicillin/sulbactam; CFZ, cefazolin; FEP, cefepime; CAZ, ceftazidime; CRO, ceftriaxone; TZP, piperacillin/tazobactam; CIP, ciprofloxacin; MEM, meropenem; TOB, tobramycin; GEN, gentamicin; SMZ, trimethoprim/sulfa.

For the 279 isolates, meropenem exerted the highest susceptibility rate (98.9%), followed by piperacillin/tazobactam (94.3%), and the last was ampicillin/sulbactam (80.6). The survival group had an obviously higher ampicillin/sulbactam and cefazolin susceptibility rate than the non-survival group (**Figure 2A**). Actually, except for ceftazidime and trimethoprim/sulfa, the susceptibility rates of all drugs were relatively higher in the survival group than in the non-survival group. Except for meropenem, the septic shock group had a higher susceptibility rate of all drugs than the non-septic shock group (**Figure 2B**).

### Effect of statins use on 28-day mortality and septic shock

In univariate analysis (**Table 1**), the hyperlipidemic group had a lower risk of death within 28 days than the non-hyperlipidemic group (OR 0.309, 95% CI 0.123-0.774, p = 0.009). However, there was no significant difference in the risk of septic shock (OR 0.664, 95% CI 0.362-1.219, p = 0.185) between the two groups (**Table 3**).

Consequently, we explored the relationship between statins use and 28-day mortality in all patients. As a result of multivariate analysis (**Table 2**), patients with statins use had a lower risk of death within 28 days than those without statins use (OR 0.220, 95% CI 0.060-0.801, p = 0.022). Similarly, no significant difference was found in the risk of septic shock between the two groups (OR 0.775, 95% CI 0.414-1.451, p = 0.424) (**Table 3**).

**Figure 3:**
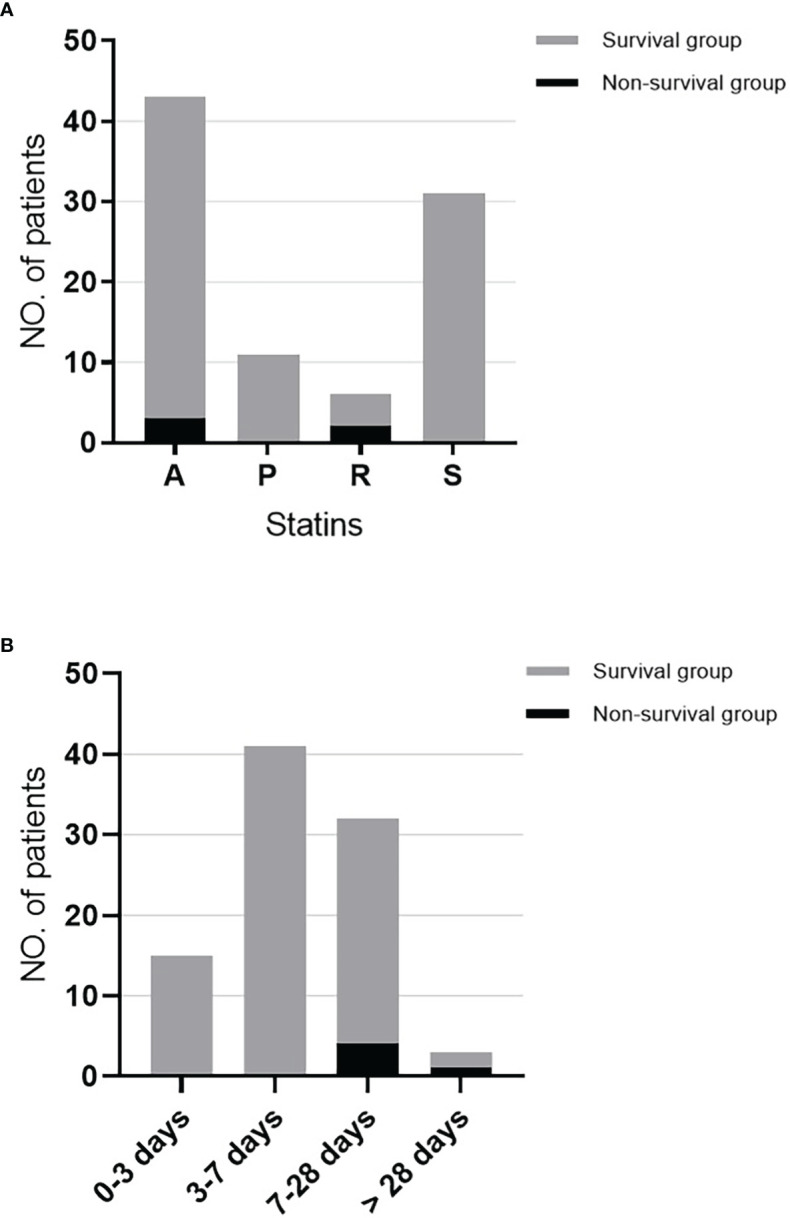
The use of statins and death within 28-day in statins use patients. **(A)** The distribution of statins categories and death within 28-day in statins use patients. **(B)** The distribution of statins duration and death within 28-day in statins use patients. A, Atorvastatin; P, Pravastatin; R, Rosuvastatin; S, Simvastatin.

There were totally 91 patients used statins which varied from Simvastatin, Pravastatin, Atorvastatin, and Rosuvastatin during the hospitalization. It was observed that only three patients using Atorvastatin and two using Rosuvastatin died within 28-day (**Figure 3A**). The statins duration time of the patients could be divided into four groups as showed in **Figure 3B**, in which four and one patient died within 28-day in group 7-28 days and group more than 28 days, respectively.

### Risk prediction model for 28-day mortality and sepsis/septic shock

**Figure 4:**
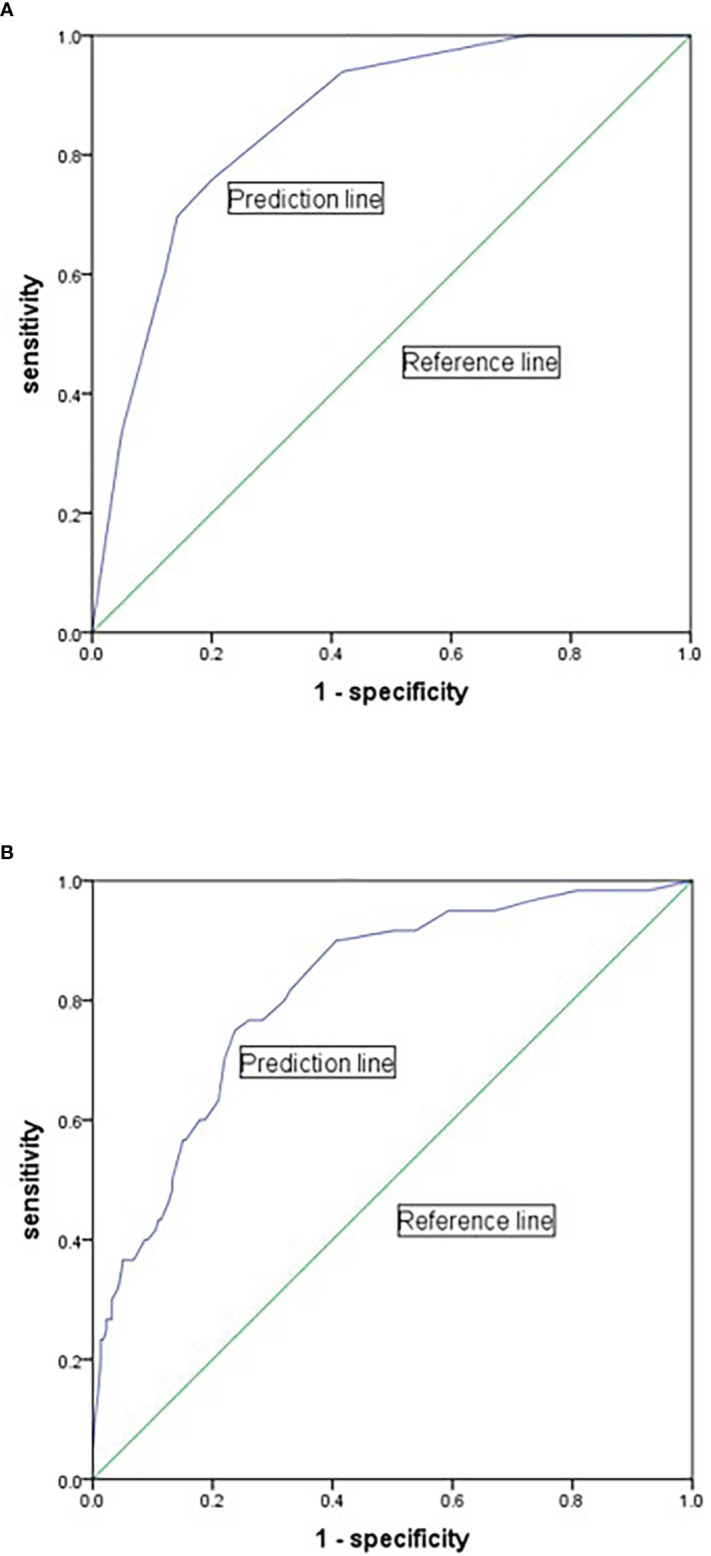
Receiver-operator characteristic curve for predicting the 28-day mortality and septic shock of BSI-KP. **(A)** The model was based on the variables of statins use, quinolones use and vasoactive agent use to predict 28-day mortality. **(B)** The model was based on the variables of gender, pulmonary disease, bleeding and coagulation disorders, respiratory failure, kidney dysfunction and hypertension for predicting septic shock.

We created a logistic regression model to predict the probability of 28-day mortality based on the results of multivariate analysis. The Omnibus test and the Hosmer-Lemeshow test demonstrated that this model was generally meaningful (p < 0.001) and had a good fit to the data (p = 0.799), respectively. ROC curve showed that the integration of quinolones uses, vasoactive agent use, statins use was good for prediction with an AUROC of 0.855 (95% CI = 0.796–0.914) (p < 0.001) (**Figure 4A**). We also established a model for evaluating the risk of septic shock and confirmed the model quality with the Omnibus test (p < 0.001) and the Hosmer-Lemeshow test (p = 1.000). ROC curve showed that the integration of gender, pulmonary disease, bleeding and coagulation disorders, respiratory failure, kidney dysfunction and hypertension could assess risk of septic-shock to some extent, with an AUROC of 0.815 (95% CI = 0.755–0.874) (p < 0.001) (**Figure 4B**).

## Discussion

It was the first time for us to investigate the risk factors of mortality in patients with BSI-KP based on the MIMIC IV database. Since a number of improvements have been made, MIMIC IV released a series of new tables, including table *microbiologyevents* that comprised of variety culture results. This study collected data from MIMIC IV v1.0, which was released on March 16th, 2021. Compared to recent research results, the 28-day mortality in our study was relatively lower (11.8%) partly because most BSI-KP isolates were carbapenem-susceptible, and the antimicrobial treatment guided by the culture result was effective. According to the data, isolates from 1/33 patients of the non-survival group were meropenem resistant. Only 3/279 patients were positive with meropenem-resistant KP in blood culture.

As results from **Table 2**, patients with hyperlipidemia and those with statins use had a lower risk of death within 28 days than those without hyperlipidemia and statins use. It was also found that hyperlipidemia patients are more likely to use statins than non-hyperlipidemia patients (**Table S2**). Then we focused on the effect of statins used in 28-day mortality. The result suggested that statins use presented no statistical difference of mortality analysis neither in hyperlipidemia patients nor in non-hyperlipidemia patients (**Table S1**), which probably attributed to the sample size and the intergroup heterogeneity. A multivariate analysis was performed to confirm the potential correlation between statins and 28-day mortality. As shown in **Table 2**, patients with statins use had a lower risk of death within 28 days than those without statin use. Therefore, statins use was an independent protective factor for the death of patients with BSI-KP.

Statins have lipid-lowering properties by inhibiting 3-hydroxy-3 methylglutaryl coenzyme A reductase, a rate-limiting enzyme in cholesterol biosynthesis. They also have anti-inflammatory and immunomodulatory effects resulting in improved endothelial function, reduced thrombogenicity, and plaque stabilization (Dobesh and Olsen, 2014). Fifteen years ago, a meta-analysis published on Lancet (Lewington et al., 2007) demonstrated that statins use could reduce coronary event rates and total stroke rates in patients with a wide range of ages and blood pressures. Researchers had focused on the functions of statins earlier, especially in patients with sepsis (Almog, 2003; Novack et al., 2006). Since then, a series of studies have been performed and showed that statin use improved the outcome of BSI patients with transplantation and cancer (Sun et al., 2009; Hsu et al., 2009; Yu et al., 2014). However, there found no evidence indicating that statins use is associated with a reduction in mortality of sepsis patients (Pasin et al., 2013; Wan et al., 2014). In our study, it was observed that statins use had a significant difference in the risk of septic shock and the 28-day mortality of patients admitted into the intensive care unit (ICU) (**Table S3**). Besides, Mehl et al. (Mehl et al., 2015) proposed that prior statin use is associated with a lower 90-day total mortality in Gram-negative BSI but not in Gram-positive BSI. However, two studies showed statin treatment in patients with *Staphylococcus aureus* bacteremia was associated with lower mortality (Lopez-Cortes et al., 2013; Smit et al., 2017). This study also found that statin use could decrease the 28-day mortality in patients with BSI-KP (**Table 2**).

The effect of statins in patients may be different for individual statins. Lee et al. (Lee et al., 2018) found that simvastatin (hazard ratio [HR], 0.72; 95% CI, 0.58-0.90) and atorvastatin (HR, 0.78; 95% CI, 0.68-0.90) were associated with improved 30-day survival, whereas rosuvastatin was not. Quellette et al. (Ouellette et al., 2015) pointed out that atorvastatin use was associated with improved mortality in septic patients compared with pre-hospital simvastatin use. In addition, a study base on patients with chronic obstructive pulmonary disease (COPD) also observed that Fluvastatin and Atorvastatin are more effective in reducing C-reactive protein and pulmonary hypertension (Lu et al., 2019). It inferred that the drug-specific effect of statins on BSI is not correlated to their lipid-lowering potency. Statins have pleiotropic effects such as anti-inflammatory, antithrombotic, and antioxidant effects and their lipid-lowering effects. In sepsis and septic shock, cytokine release from endothelial cells, procoagulant molecules, and thrombocyte production are encouraged. Statins act on sepsis by providing vascular relaxation in the endothelium and reducing the expression of adhesion molecules and cytokines (Goncuoglu et al., 2021).

On the other hand, Pawar et al. (Pawar et al., 2018) found that continued statins use for at least two days after admission provided a survival benefit among bacteremia patients. Similar to our study, about 77% (21/91) of patients continued using statins for more than two days. Thus, more laboratory and clinical data are encouraged, and randomized controlled trials will be needed to define the role of statins in BSI patients.

Based on the binary logistic regression results, we built two ROC curves to predict the risk of 28-day mortality and septic shock in BSI-KP patients. In the model for 28-day mortality, the variable age was considered as a confounding factor because of no clinical significance and was excluded (**Figure 4A**). Quinolones use was regarded as a protective factor mainly attributed to the majority isolates were sensitive to ciprofloxacin (**Figure 2**). There were two main reasons for the protective effect of quinolones. One is the combination of drugs in the process of anti-infection treatment, and the other is that the utilization rate of carbapenems (20.8%) is lower than quinolones (57.7%). By contrast, the effectiveness of variable gender in the model for septic shock was still preserved since it was not determined whether males were less likely to suffer from septic shock (**Figure 4B**). As a result, both of the models had good value for prediction.

This study has some limitations. First, the data were obtained from a single institution during a limited study period, and thus, the results may not be widely representative or generalizable. Second, the data was insufficient to detail the effect of statins use on the 28-day mortality of patients. Third, since it was a retrospective study, the confounders still exist, such as patients’ comorbidities, and the selection bias may influence the results. With the current knowledge, more studies are needed to confirm the use of statins to benefit from their pleiotropic effects in the treatment of BSI-KP patients.

## Conclusion

The use of statins could decrease the risk of 28-day mortality in patients of BSI-KP. The risk factor-based prediction model provided evidence for drug treatment in BSI-KP patients. Paying more attention to the treatment strategy will be an optimal way to improve outcomes in clinical practice.

## Data availability statement

The original contributions presented in the study are included in the article/**Supplementary Material**. Further inquiries can be directed to the corresponding author.

## Ethics statement

Ethical review and approval was not required for the study on human participants in accordance with the local legislation and institutional requirements. Written informed consent for participation was not required for this study in accordance with the national legislation and the institutional requirements.

## Author contributions

QX designed the study and extracted the data. BZ and QX analyzed the data and drafted the manuscript. YX and BZ supervised the study and revised the manuscript. PS contributed to the data analyzation and the figure presentation. All authors contributed to the article and approved the submitted version.
