# Supplementary material for: Protective efficacy of statins in patients with Klebsiella pneumoniae bloodstream infection
Source: Front Cell Infect Microbiol. 2023 Jan 5;12:1087701. doi: 10.3389/fcimb.2022.1087701 (PMC9849249; doi:10.3389/fcimb.2022.1087701)
Supplement: Supplementary file 1 [file Table_1.docx]

**Supplemental materials**

**Table S1. The statins used in the analysis for 28-day mortality (A) and sepsis/septic shock (B) in hyperlipidemia patients and non-hyperlipidemia patients with BSI-KP.**

| **(A) Statins use** | Total | Non-survival group | Survival  group | P-value | OR (95% CI) |
| --- | --- | --- | --- | --- | --- |
| In hyperlipidemia | 53 (48.6) | 2 (33.3) | 51 (49.5) | 0.679 | 0.510 (0.089-2.907) |
| In non-hyperlipidemia | 38 (22.4) | 3 (11.1) | 35 (24.5) | 0.206 | 0.386 (0.109-1.359) |

OR: odds ratio; CI: confidence interval.

| **(B) Statins use** | Total | Septic shock  group | Non-septic shock group | P-value | OR (95% CI) |
| --- | --- | --- | --- | --- | --- |
| In hyperlipidemia | 53 (48.6) | 9 (47.4) | 44 (48.9) | 0.904 | 0.941 (0.349-2.534) |
| In non-hyperlipidemia | 38 (22.4) | 8 (19.5) | 30 (23.3) | 0.616 | 0.800 (0.334-1.917) |

OR: odds ratio; CI: confidence interval.

**Table S2. The distribution of statins uses and hyperlipidemia in BSI-KP patients.**

| **NO. of patients** |  | **Statins use** | |  |
| --- | --- | --- | --- | --- |
|  |  | Y | N | Total |
| **Hyperlipidemia** | Y | 53 | 56 | 109 |
|  | N | 38 | 132 | 170 |
|  | total | 91 | 188 | 279 |

Y: yes; N: no.

**Table S3. Predictors for 28-day mortality in 96 patients admitted into ICU by univariate and multivariate analysis**

|  | **Univariate** | |  | **Multivariate** | |
| --- | --- | --- | --- | --- | --- |
| **Variables, n (%)** | OR (95%CI) | P-value |  | OR (95%CI) | P-value |
| Age, years (IQR) | - | 0.655 |  | 0.976 (0.911-1.045) | 0.485 |
| Male | 2.581 (0.767-8.691) | 0.117 |  | 2.333 (0.505-10.777) | 0.278 |
| Pulmonary disease | 3.486 (1.036-11.738) | 0.036 |  | 1.727 (0.349-8.542) | 0.503 |
| Hypertension | 0.303 (0.096-0.955) | 0.035 |  | 0.690 (0.141-3.376) | 0.647 |
| Hyperlipidemia | 0.282 (0.075-1.067) | 0.051 |  | 0.448 (0.066-3.508) | 0.412 |
| Respiratory failure | 7.578 (2.328-24.666) | 0.001 |  | 3.110 (0.557-17.374) | 0.196 |
| Kidney dysfunction | 3.729 (0.986-14.100) | 0.042 |  | 0.951 (0.167-5.412) | 0.955 |
| SAPSII | - | <0.001 |  | 0.966 (0.913-1.021) | 0.218 |
| SOFA | - | 0.053 |  | 1.005 (0.766-1.320) | 0.970 |
| Vasoactive agent using | 20.294 (2.555-161.183) | <0.001 |  | 7.950 (0.752-84.090) | 0.085 |

OR: odds ratio; CI: confidence interval; SAPSII: Simplified Acute Physiology Score II; SOFA: Sequential Organ Failure Assessment.
